# A Sparse-Based Off-Grid DOA Estimation Method for Coprime Arrays

**DOI:** 10.3390/s18093025

**Published:** 2018-09-10

**Authors:** Weijian Si, Fuhong Zeng, Changbo Hou, Zhanli Peng

**Affiliations:** College of Information and Communication Engineering, Harbin Engineering University, Harbin 150001, China; swj0418@263.net (W.S.); fuhongzeng@gmail.com (F.Z.); zhanlipeng9@gmail.com (Z.P.)

**Keywords:** DOA estimation, coprime arrays, sparse-based methods, off-grid, grid biases

## Abstract

Recently, many sparse-based direction-of-arrival (DOA) estimation methods for coprime arrays have become popular for their excellent detection performance. However, these methods often suffer from grid mismatch problem due to the discretization of the potential angle space, which will cause DOA estimation performance degradation when the target is off-grid. To this end, we proposed a sparse-based off-grid DOA estimation method for coprime arrays in this paper, which includes two parts: coarse estimation process and fine estimation process. In the coarse estimation process, the grid points closest to the true DOAs, named coarse DOAs, are derived by solving an optimization problem, which is constructed according to the statistical property of the vectorized covariance matrix estimation error. Meanwhile, we eliminate the unknown noise variance effectively through a linear transformation. Due to finite snapshots effect, some undesirable correlation terms between signal and noise vectors exist in the sample covariance matrix. In the fine estimation process, we therefore remove the undesirable correlation terms from the sample covariance matrix first, and then utilize a two-step iterative method to update the grid biases. Combining the coarse DOAs with the grid biases, the final DOAs can be obtained. In the end, simulation results verify the effectiveness of the proposed method.

## 1. Introduction

Direction of arrival (DOA) estimation of multiple far-field narrowband sources is a vital and interesting topic in a signal processing field, which has wide application in many areas such as radar, sonar, radio astronomy and wireless communications, etc. [1,2,3,4]. As we all know, the DOA estimation problem can be solved efficiently by classical subspace-based methods such as multiple signal classification (MUSIC) [5] and estimation of signal parameters via rotational invariance technique (ESPRIT) [6] in conditions where the receiving arrays are mostly uniform arrays, such as uniform linear array (ULA) [7], uniform rectangular array (URA) [8] and uniform circular array (UCA) [9]. The inter-element spacing of these receiving arrays is required to be less than or equal to the half-wavelength of received signal. Therefore, the detection performance of these DOA estimation methods is greatly confined by the number of physical sensors. For example, a maximum of M−1 sources can be detected when these algorithms are applied to a ULA with *M* sensors. Accordingly, more sensors are needed in order to detect more sources and acquire the desirable estimation accuracy. However, a large number of sensors will increase the hardware cost and the difficulty of array calibration in practical applications. To overcome this challenge, some non-uniform array geometries, called sparse arrays, have been proposed, such as minimum redundancy arrays (MRAs) [10], nested arrays (NAs) [11,12] and coprime arrays (CPAs) [13,14,15]. Though MRAs can obtain more degrees of freedom (DOFs) through constructing an augmented covariance matrix, they have no closed form expressions for the optimal array configurations as well as the achievable DOFs for an arbitrary number of sensor elements. Unlike MRAs, the NAs with $O\left(N\right)$ physical sensors can achieve $O\left(N^{2}\right)$ DOFs and they have closed form expressions for the optimal sensors positions. However, mutual coupling arising from the dense subarray of NAs may exist, which will cause the DOA estimation performance degradation. To circumvent this problem, coprime arrays which can detect $O\left(MN\right)$ sources with only $O\left(M+N\right)$ physical sensors were proposed in [13], where *M* and *N* are coprime integers.

When the incident signals are received by the CPAs, a virtual array with extended aperture can be achieved by vectorizing the receiving data covariance matrix. Thus, more DOFs than sensors can be obtained. In order to exploit the extended DOFs for DOA estimation, many algorithms have been proposed. The spatial smoothing MUSIC (SS-MUSIC) algorithm for comprime arrays was first proposed in [16], which has robust estimation performance and can detect more sources than sensors in coarray domain. Unfortunately, it needs to know the number of sources in advance. To this end, a MUSIC-like subspace method was proposed in [17], in which the number of sources is a by-product of low rank denoising stage. In [18], a novel coarray ESPRIT-based DOA estimation algorithm was proposed to balance the contradiction between estimation performance and computation complexity. However, all of the aforementioned subspace methods can only utilize the consecutive difference coarray. Additionally, due to the application of spatial smoothing technique [19], half of the continuous DOFs are lost, which will result in a significant degradation of detection performance. Accordingly, in order to maximize the utilization of extended DOFs, some sparse representation methods for coprime arrays [20,21,22] were proposed, which can be directly applied to derive the signal power vector without requiring decorrelation and covariance matrix rank recovery operation. Generally speaking, the extended virtual array aperture can be fully used for DOA estimation in these sparse-based methods. Consequently, they can detect more sources than those subspace methods and own better detection performance. On the basis of [16], a least absolute shrinkage and selection operator (LASSO) algorithm with extended coprime array geometry [23] was proposed, which can provide more DOFs than [16]. Now, this extended coprime array geometry is used very widely due to its excellent performance. Though there are so many advantages for these sparse-based methods, there is still a disadvantage that they need to discretize the angle space and preset enough grid points. The precondition for estimating the DOAs successfully is that the locations of the sources must fall on the predefined grid points. However, in practical situations, no matter how fine the grids are, it is impossible that the sources always lie on the predefined grid points. Thus, the off-grid sources may cause dictionary mismatch problems. As a result, the recovery performance of these methods will be deteriorated.

In recent years, lots of methods [24,25,26,27,28,29,30,31,32] have been proposed for solving the off-grid problems. Zhang et al. [24] presented a block-sparse Bayesian algorithm to solve the grid mismatch problem, in which the noise variance can be normalized to 1 and thus its effect on the estimation performance can be reduced. In references [25,26], the joint sparsity between the original signals and the grid biases was used to solve the off-grid problem. In a previous study [27], a gridless sparse method was developed for off-grid/continuous DOA estimation by minimizing the reweighted atomic norm. In reference [28], by first-order Lagrange approximation, an off-grid signal model with approximate dictionary matrix is established. Then, the grid biases are derived in sparse Bayesian learning approach by using the joint sparsity between different snapshots of the received signal. In reference [29], a root sparse Bayesian learning method was proposed, in which there is no need to construct off-grid model. The grid points are iteratively updated by finding the roots of a polynomial. Based on Dai et al. [29], a grid evolution direction of arrival (GEDOA) estimation method [30], which includes two sub-processes, i.e., learning process and fission process, was proposed to solve the problem of more than one DOA in one initial grid interval and speed up the method proposed in reference [29]. Wu et al. [31] proposed two iterative methods, both of which update the signal power vector and off-grid biases alternately. Wang et al. [32] proposed a real-valued formulation of covariance vector-based relevance vector machine (CVRVM) technique, which is implemented in a real domain and has low computation complexity. However, these methods are all applied on the traditional uniform linear array and they do not utilize the increased DOFs provided by the difference coarray of coprime arrays. Thus, they cannot detect more sources than sensors. As mentioned before, virtual array aperture extension can be described effectively by the vectorized covariance matrix model. However, it is not rational to directly apply most sparse-based methods to this model as those methods are usually designed to work in the raw data domain. Due to the superiority of coprime arrays, some off-grid DOA estimation methods have been proposed for coprime arrays. In reference [33], an off-grid DOA estimation method based on the framework of sparse Bayesian learning was proposed for coprime arrays. The predefined grid points are directly updated by iteratively decreasing a surrogate function majorizing the given objective function. Sun et al. [34] proposed an iterative method to obtain the final DOA estimation by gradually amending the offset vector with introducing a convex function majorizing the objective function. Both of the methods in references [33,34] need to use the gradient descent method, in which the gradient descent coefficient is difficult to choose. If the gradient descent coefficient is too large, the DOA estimation accuracy will be reduced. On the contrary, if the gradient descent coefficient is too small, the convergence speed of the algorithms will be limited.

To deal with the above difficulties, a sparse-based off-grid DOA estimation method for coprime arrays is proposed in this paper. The proposed method includes two processes, i.e., coarse estimation process and fine estimation process. In the coarse estimation process, we consider the estimation error caused by finite snapshots effect and remove the redundant elements in the covariance vector (i.e., vectorized covariance matrix). In addition, we circumvent estimating the noise variance by using a linear transformation to remove the noise-related part in the covariance vector. This is due to the fact that the noise variance is not so easy to evaluate especially under the underdetermined scenarios. Through a series of linear transformations, the covariance matrix estimation error can be normalized to an identity matrix. Then, according to the statistical property of the vectorized covariance matrix estimation error, DOA estimation can be cast as a problem of recovering a nonnegative sparse vector with an extended steering vector. The grid points corresponding to the non-zero elements in the recovered sparse vector are coarse DOA estimation results for the true signals, which are taken as the predefined grid points required for the fine estimation process. In practical applications, the number of snapshots is finite, which will cause the sample covariance matrix deviating from the actual covariance matrix largely [35]. Therefore, in the fine estimation process, the sample covariance matrix is modified firstly and then the grid biases are derived by applying a two-step iterative technique to the vectorized modified covariance matrix. The final estimated DOAs can be obtained by integrating the coarse DOA estimation results and the grid biases.

The rest of the paper is organized as follows. The far-field narrowband signal model for coprime arrays is briefly introduced in Section 2. Section 3 formulates the proposed sparse DOA estimation method. Section 4 provides several numerical simulation results to show the superior estimation performance for the proposed method and Section 5 concludes this paper.

Notations: In this paper, italic lower-case (upper-case) bold characters are used to denote vectors (matrices). In particular, $I_{K}$ denotes the K×K identity matrix. Sets are denoted by capital letters in blackboard boldface. Specifically, the symbols R, C and Z represent the sets of real numbers, complex numbers and integers, respectively. ${\left(\cdot \right)}^{*}$, ${\left(\cdot \right)}^{T}$, ${\left(\cdot \right)}^{H}$ and ${\left(\cdot \right)}^{-1}$ imply conjugate, transpose, conjugate transpose and inverse, respectively. $\mathrm{vec}\left(\cdot \right)$ denotes the vectorization operator. The symbols ∘, ⊙ and ⊗ represent Hadamard product, Khatri–Rao product and Kronecker product, respectively. $\mathrm{diag}\left(a\right)$ stands for a diagonal matrix whose diagonal elements are taken from the given vector a, while $\mathrm{diag}\left(A\right)$ represents a column vector whose elements are the diagonal elements of the given matrix A. $ℜ\left(\cdot \right)$ means taking the real part of a complex value. The $l_{1}$-norm and $l_{2}$-norm are respectively denoted by ${∥\cdot ∥}_{1}$ and ${∥\cdot ∥}_{2}$. $\mathrm{tr}\left(\cdot \right)$ is the trace operator. $E\left[\cdot \right]$ denotes the statistical expectation operator. $CN\left(a,B\right)$ denotes a complex Gaussian distribution with mean vector a and covariance matrix B.

## 2. Signal Model

**Figure 1:**
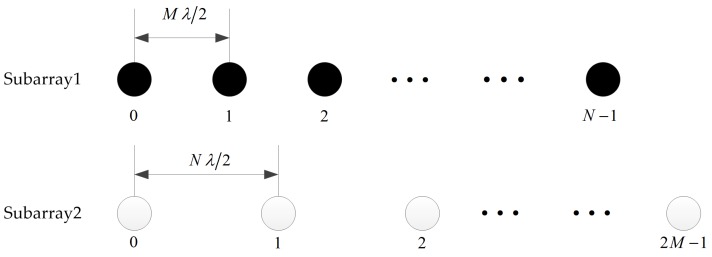
The geometry of the two subarrays in the coprime arrays.

As illustrated in Figure 1, consider the coprime arrays which are composed of two uniform linear subarrays. One includes *N* sensors with inter-element spacing MλMλ22, while the other owns 2M sensors with inter-element spacing NλNλ22, where λ is the wavelength of the received signal. As described in Section 1, *M* and *N* are coprime integers. Without loss of generality, we assume that *M* is less than *N*. The two subarrays share the zeroth sensor, which is also the reference sensor. Thus, the total number of sensors in the coprime arrays is 2M+N−1, which are located at

(1) L=nMλλ22n∈Z,0≤n≤N−1∪mNλλ22m∈Z,0≤m≤2M−1=liλλ22,i=1,2,⋯,2M+N−1,

where L is sorted in ascending order with $l_{1}=0$ and $l_{2M+N-1}=\left(2M-1\right)N$.

Suppose *K* far-field narrowband signals are incident on the coprime arrays from directions $\left(\theta_{1},\theta_{2},\cdots \theta_{K}\right)$, where $\theta_{k}\in \left[-\frac{\pi }{2},\frac{\pi }{2}\right],k=1,2,\cdots ,K$ is the direction of the *k*-th signal. Then, the received signal at time *t* can be expressed as

(2) $$x\left(t\right)=\sum_{k=1}^{K}a\left(\theta_{k}\right)s_{k}\left(t\right)+n\left(t\right)=As\left(t\right)+n\left(t\right).$$

In Equation (2), $s\left(t\right)={\left[s_{1}\left(t\right),s_{2}\left(t\right),\cdots ,s_{K}\left(t\right)\right]}^{T}\in C^{K\times 1}$ is the signal vector at time $t\left(0\leq t\leq T\right)$, which is independent from the signal vectors at other time instances. *T* is the number of snapshots. $n\left(t\right)={\left[n_{1}\left(t\right),n_{2}\left(t\right),\cdots ,n_{2M+N-1}\left(t\right)\right]}^{T}$ is the noise vector at time *t*, the elements of which are assumed to be temporally and spatially independent and identically distributed (i.i.d.) random variables which satisfy the complex Gaussian distribution with zero mean and variance $\sigma_{}^{2}$. When the number of sampled snapshots is sufficiently large, the signal vectors can be assumed to be independent from the noise vectors. $A=\left[a\left(\theta_{1}\right),a\left(\theta_{2}\right),\cdots ,a\left(\theta_{K}\right)\right]\in C^{\left(2M+N-1\right)\times K}$ is the array manifold matrix, where $a\left(\theta_{k}\right)$ is the steering vector for source *k* and it can be written as

(3) $$a\left(\theta_{k}\right)={\left[e^{j\pi l_{1}\sin\theta_{k}},e^{j\pi l_{2}\sin\theta_{k}},\cdots ,e^{j\pi l_{2M+N-1}\sin\theta_{k}}\right]}^{T}.$$

Since the number of sampled snapshots is finite in practical applications, the sample covariance matrix can be approximated as

(4) $${\hat{R}}_{xx}=\frac{1}{T}\sum_{t=1}^{T}x\left(t\right)x^{H}\left(t\right).$$

## 3. Proposed Sparse-Based Off-Grid Direction of Arrival Estimation Method

For the sake of solving the problem that the incident signals are not always located on the predefined grid points, an off-grid DOA estimation method is proposed in this section. The method includes two parts: coarse estimation process and fine estimation process. The coarse estimation process is to determine the nearest grid point to the direction of each incident signal, while the fine estimation process is to find the bias between the nearest grid point and the true DOA.

### 3.1. Coarse Estimation Process

One of the tasks for the coarse estimation process is to estimate the positions of the grid points closest to the incident signals. In addition, it also provides the parameters needed for the fine estimation process.

Under the situations where the number of sampled snapshots is sufficiently large, the received data covariance matrix can be denoted as

(5) $$R_{xx}=E\left[x\left(t\right)x^{H}\left(t\right)\right]=AR_{s}A^{H}+\sigma_{}^{2}I_{2M+N-1}.$$

Here, $R_{s}=E\left[s\left(t\right)s^{H}\left(t\right)\right]=\mathrm{diag}\left(\sigma_{1}^{2},\sigma_{2}^{2},\cdots ,\sigma_{K}^{2}\right)$ is the theoretical signal covariance matrix and $\sigma_{k}^{2}\left(k=1,2,\cdots ,K\right)$ denotes the power of the *k*-th signal. By vectorizing the covariance matrix $R_{xx}$, the covariance vector can be obtained as

(6) $$r=\mathrm{vec}\left(R_{xx}\right)\;=\tilde{A}p+\sigma^{2}1_{n},$$

where $\tilde{A}=\left[a^{*}\left(\theta_{1}\right)⊗a\left(\theta_{1}\right),a^{*}\left(\theta_{2}\right)⊗a\left(\theta_{2}\right),\cdots ,a^{*}\left(\theta_{K}\right)⊗a\left(\theta_{K}\right)\right]$, $p={\left[\sigma_{1}^{2},\sigma_{2}^{2},\cdots ,\sigma_{K}^{2}\right]}^{T}$ and $1_{n}\;=\;\mathrm{vec}\left(I_{2M+N-1}\right)={\left[e_{1}^{T},e_{2}^{T},\cdots ,e_{2M+N-1}^{T}\right]}^{T}$. $e_{i}$ denotes a column vector whose elements are all zeros except that the *i*-th element is one. r can be seen as the received data from the virtual array with extended array aperture, where the virtual sensors are located at

(7) $$D=\left\{d_{i}-d_{j}\left|d_{i},d_{j}\in L,1\leq i,j\leq 2M+N-1\right.\right\}.$$

The sample covariance matrix is as described in Equation (4). Owing to the finite snapshots, the estimation error between the actual and sample covariance matrix needs to be considered. Thus, vectorizing the sample covariance matrix, we can get

(8) $$\hat{r}=\mathrm{vec}\left({\hat{R}}_{xx}\right)=\tilde{A}p+\sigma^{2}1_{n}+\varepsilon ,$$

where ε is the vectorized covariance matrix estimation error and it satisfies an asymptotic Gaussian distribution [36,37]

(9) $$\varepsilon =\mathrm{vec}\left({\hat{R}}_{xx}^{}-R_{xx}\right)∼CN\left(0,\frac{1}{T}R_{xx}^{T}⊗R_{xx}^{}\right).$$

The number of extended virtual sensors in Equation (6) is ${\left(2M+N-1\right)}^{2}$, while the number of non-redundant virtual sensors is 3MN+M−N. These duplicate virtual sensors only provide unnecessary redundant information. Thus, for the sake of saving computation complexity, we remove the repeated rows of $\tilde{A}$ corresponding to the redundant virtual sensors and sort the remaining rows to construct a new virtual array manifold matrix B. Similarly, the same transformation is performed on $\hat{r}$ to obtain the corresponding received data vector z, i.e.,

(10) $$z=Bp+\sigma^{2}e+\bar{\varepsilon },$$

where e is a column vector whose elements are all zeros except that the element at the center is one. It is obvious that $\bar{\varepsilon }$ is different from ε. The relationship between $\bar{\varepsilon }$ and ε will be given later.

We define $\bar{D}$ as the set, the elements of which are the locations of the virtual sensors corresponding to array manifold matrix B. It can be directly derived by subtracting the duplicates from the difference D and sorting the remaining entries in ascending order and can be denoted as

(11) D¯=d¯iλλ22,i=1,2,⋯,3MN+M−N,

where ${\bar{d}}_{1}=-\left(2M-1\right)N$ and ${\bar{d}}_{3MN+M-N}=\left(2M-1\right)N$. Let $B\;=\;\left[b\left(\theta_{1}\right),b\left(\theta_{2}\right),\cdots ,b\left(\theta_{K}\right)\right]\in C^{\left(3MN+M-N\right)\times K}$ and $b\left(\theta_{k}\right)$ is the virtual steering vector for the *k*-th signal with the *i*-th element $e^{j\pi {\bar{d}}_{i}\sin\theta_{k}}$, ${\bar{d}}_{i}\in \bar{D}$.

According to [33], we can get the relationship between ε and $\bar{\varepsilon }$:

(12) $$\varepsilon \;=\;\left(\Phi ⊗\Phi \right)G\Psi^{-1}\bar{\varepsilon }.$$

For the convenience of notation expression, let $F=\left(\Phi ⊗\Phi \right)G\Psi^{-1}$ in Equation (12), where $\Phi \in {\left\{0,1\right\}}^{\left(2M+N-1\right)\times \left[\left(2M-1\right)N+1\right]}$ is a selection matrix, the *i*-th row of which contains all zeros but a single one at the $\left(l_{i}+1\right)$-th position. $\Psi \in {\left\{0,1\right\}}^{\left(3MN+M-N\right)\times \left[2N\left(2M-1\right)+1\right]}$ is also a selection matrix, the elements of which are all zeros in the *i*-th row except a single one at the $\left({\bar{d}}_{i}\;+\;2MN-N+1\right)$-th position. $G\in R^{{\left[N\left(2M-1\right)+1\right]}^{2}\times \left[2N\left(2M-1\right)+1\right]}$ can be denoted as

(13) $$G=\left[\begin{array}{lll} 0_{\left[N\left(2M-1\right)+1\right]\times \left[N\left(2M-1\right)\right]} & & I_{N\left(2M-1\right)+1}\\ 0_{\left[N\left(2M-1\right)+1\right]\times \left[N\left(2M-1\right)-1\right]} & I_{N\left(2M-1\right)+1} & 0_{\left[N\left(2M-1\right)+1\right]\times 1}\\ \vdots & \vdots & \vdots \\ I_{N\left(2M-1\right)+1} & & 0_{\left[N\left(2M-1\right)+1\right]\times \left[N\left(2M-1\right)\right]} \end{array}\right].$$

Let $\vartheta \;=\;\left(\vartheta_{1},\vartheta_{2},\cdots ,\vartheta_{L}\right)$ be the grid points sampled from $\left[-\frac{\pi }{2},\frac{\pi }{2}\right]$ with appropriate step size, where *L* is the total number of grid points and L≫K in general. Then, the virtual received signal model in Equation (10) can be sparsely represented as

(14) $$z=\bar{B}\bar{p}+\sigma^{2}e+\bar{\varepsilon },$$

where $\bar{B}\;=\;\left[b\left(\vartheta_{1}\right),b\left(\vartheta_{2}\right),\cdots ,b\left(\vartheta_{L}\right)\right]\in C^{\left(3MN+M-N\right)\times L}$ is the overcomplete dictionary matrix and $\bar{p}$ is the extension of p with zeros filling in the positions where no signals stay in.

As mentioned before, $\sigma^{2}e$ has only one non-zero element at its own center. Therefore, to avoid calculating the noise variance, we remove the corresponding row in the received data vector z by a linear transformation, so that Equation (14) can be transformed to a noiseless received signal model, which can be expressed as

(15) $$\bar{z}=Jz=J\bar{B}\bar{p}+J\bar{,}\varepsilon ,$$

where $J\in R^{\left(3MN+M-N-1\right)\times \left(3MN+M-N\right)}$ can be denoted as

(16) J=I3MN+M−N−13MN+M−N−12203MN+M−N−13MN+M−N−122×3MN+M−N−13MN+M−N−122+103MN+M−N−13MN+M−N−122×3MN+M−N−13MN+M−N−122+1I3MN+M−N−13MN+M−N−122.

Combining Equations (9) with (12) and (15), we can get

(17) Jε¯∼CN0,JF−1RxxT⊗RxxJF−1TJF−1RxxT⊗RxxJF−1TTT.

From Equations (15) and (17), we can derive

(18) $$W^{-1/2}\left(\bar{z}-J\bar{B}\bar{p}\right)∼CN\left(0,I_{3MN+M-N-1}\right),$$

where $W=JF^{-1}\left(R_{xx}^{T}⊗R_{xx}^{}\right){\left(JF^{-1}\right)}^{T}/T$ and $W^{-1/2}$ is the Hermitian square root of $W^{-1}$. Then,

(19) $${∥W^{-1/2}\left(\bar{z}-J\bar{B}\bar{p}\right)∥}_{2}^{2}∼As\chi^{2}\left(3MN+M-N-1\right).$$

Here, $As\chi^{2}\left(3MN+M-N-1\right)$ denotes asymptotic chi-square distribution with $\left(3MN+M-N-1\right)$ DOFs. Thus, coarse DOA estimation results can be derived by solving the optimization problem:

(20) $$\min_{\bar{p}\geq 0}{∥\bar{p}∥}_{1}\;\;\;\;\;\;\;\;\;\;\;\;s.t.\;\;\;\;\;\;\;\;\;\;\;\;\;\;\;\;{∥{\hat{W}}^{-1/2}\left(\bar{z}-J\bar{B}\bar{p}\right)∥}_{2}^{}\leq \xi ,$$

where $\hat{W}=JF^{-1}\left({\hat{R}}_{xx}^{T}⊗{\hat{R}}_{xx}^{}\right){\left(JF^{-1}\right)}^{T}/T$ is the estimation of W. ξ can be uniquely determined by $\xi \;=\;\sqrt{chi2inv\left(1-\beta ,3MN+M-N-1\right)}$. According to experience, β is usually set as $10^{-4}$.

After attaining $\bar{p}$, the grid points corresponding to the *K* maximum values of $\bar{p}$ are the coarse DOA estimation results, called coarse DOAs, which are denoted as $\theta_{coarse}\;=\;\left({\hat{\theta }}_{1}^{\left(1\right)},{\hat{\theta }}_{2}^{\left(1\right)},\cdots ,{\hat{\theta }}_{K}^{\left(1\right)}\right)$.

### 3.2. Fine Estimation Process

The following is the fine estimation process where the grid biases can be derived according to the coarse DOAs. First of all, we expand the sample covariance matrix ${\hat{R}}_{xx}$ in Equation (4) as

(21) $$\begin{array}{ll} {\hat{R}}_{xx} & \;=\;\frac{1}{T}\sum_{t=1}^{T}\left(As\left(t\right)+n\left(t\right)\right){\left(As\left(t\right)+n\left(t\right)\right)}^{H}\\ & \;=\;A\left\{\frac{1}{T}\sum_{t=1}^{T}s\left(t\right)s^{H}\left(t\right)\right\}A^{H}+\frac{1}{T}\sum_{t=1}^{T}n\left(t\right)n^{H}\left(t\right)\\ & +A\left\{\frac{1}{T}\sum_{t=1}^{T}s\left(t\right)n^{H}\left(t\right)\right\}+\left\{\frac{1}{T}\sum_{t=1}^{T}n\left(t\right)s^{H}\left(t\right)\right\}A^{H}. \end{array}$$

Comparing (5) with (21), we can easily find that the expansion of ${\hat{R}}_{xx}$ in (21) contains four terms while $R_{xx}$ in (5) only contains two. The first two terms in (21) can be viewed as the estimates of $R_{xx}$, which correspond to the signal and noise components, respectively. The last two terms in (21) can be seen as the estimates of the correlation parts between signal and noise vectors. Generally, the signal vector $s\left(t\right)$ is assumed to be independent from the noise vector $n\left(t\right)$. Therefore, when the number of snapshots is sufficiently large, the last two terms in (21) are equal to zeros in theory. However, in practical applications, the sampled snapshots available are finite so that the undesirable correlation terms between signal and noise vectors exist, which may have significant values. The existence of these correlation terms will cause the estimate of the received data covariance matrix deviating from the actual one and further result in DOA estimation performance degradation. Accordingly, in order to improve the DOA estimation performance, we should estimate and remove these correlation terms first to construct a new modified sample covariance matrix.

From the signal model (2), we can derive the estimate of signal vector by minimizing the residual between observations and estimators, i.e.,

(22) $$\hat{s}\left(t\right)=\arg\min_{s}{∥x\left(t\right)-\hat{A}s\left(t\right)∥}_{2}^{2}.$$

Here, $\hat{A}=\left[a\left({\hat{\theta }}_{1}^{\left(1\right)}\right),a\left({\hat{\theta }}_{2}^{\left(1\right)}\right),\cdots ,a\left({\hat{\theta }}_{K}^{\left(1\right)}\right)\right]\in C^{\left(2M+N-1\right)\times K}$ is the new estimated array manifold matrix based on the coarse DOA estimation results with steering vector $a\left({\hat{\theta }}_{k}^{\left(1\right)}\right)\;=\;{\left[e^{j\pi l_{1}\sin{\hat{\theta }}_{k}^{\left(1\right)}},e^{j\pi l_{2}\sin{\hat{\theta }}_{k}^{\left(1\right)}},\cdots ,e^{j\pi l_{2M+N-1}\sin{\hat{\theta }}_{k}^{\left(1\right)}}\right]}^{T}$ for source *k*, where $l_{1},l_{2},\cdots ,l_{2M+N-1}\in L$.

Using the Least Square (LS) method to solve the optimization problem in (22), we can obtain the estimate of signal vector $s\left(t\right)$ as

(23) $$\hat{s}\left(t\right)\;=\;{\left({\left(\hat{A}{\hat{A}}^{H}\right)}^{-1}\hat{A}\right)}^{H}x\left(t\right).$$

Then, the estimate of noise vector $n\left(t\right)$ can be represented as

(24) $$\hat{n}\left(t\right)=x\left(t\right)-\hat{A}\hat{s}\left(t\right)=P_{\hat{A}}^{⊥}x\left(t\right),$$

where $P_{\hat{A}}^{}\;=\;\hat{A}{\left({\left(\hat{A}{\hat{A}}^{H}\right)}^{-1}\hat{A}\right)}^{H}$ is an estimation for the projection matrix of signal component and $P_{\hat{A}}^{⊥}\;=\;I-P_{\hat{A}}^{}$ is an estimation for the projection matrix of noise component. Combining Equations (23) with (24), the third term in (21) can be written as

(25) $$\begin{array}{c} V\;=\;\hat{A}\left\{\frac{1}{T}\sum_{t=1}^{T}\hat{s}\left(t\right){\hat{n}}^{H}\left(t\right)\right\}=P_{\hat{A}}^{}{\hat{R}}_{xx}{\left(P_{\hat{A}}^{⊥}\right)}^{H}. \end{array}$$

The fourth term in (21) is the conjugate transposition of the third one. Thus, the modified sample covariance matrix can be expressed as

(26) $$\tilde{R}={\hat{R}}_{xx}-\gamma \left(V+V^{H}\right),$$

where γ is the scale factor with a value between zero and one [35]. The value of γ depends on the estimation accuracy of the undesirable correlation terms. Ideally, γ should be equal to one when there are no errors in the estimates of the correlation terms. However, estimation errors are inevitable. Therefore, γ takes a value close to one when V in (25) has a small estimation error and a small value conversely if the estimate of V is inaccurate. The modified covariance vector $\tilde{r}$ can be generated by vectorizing $\tilde{R}$ in (26). By extracting the entries in positions corresponding to the set $\bar{D}$ from $\tilde{r}$, we can attain the covariance vector $\tilde{z}$ without redundant information. Here, the extended virtual array is consistent with that in (11).

Generally, no matter how dense the grid points are, the true signals are impossible to always fall on the predefined grid points. Therefore, it is necessary to introduce off-grid problem for improving the DOA estimation performance. The coarse DOAs are the grid points closest to the true signals. Let $\delta_{k}$ denote the grid bias for the *k*-th signal. Then, the grid biases for all *K* narrowband signals can be expressed as $\Delta =\mathrm{diag}\left(\delta \right)$, where $\delta \;=\;{\left[\delta_{1},\delta_{2},\cdots ,\delta_{K}\right]}^{T}$. The interval between two adjacent grid points in the coarse estimation process is $r_{g}$, which determines the range of the grid bias is $\left[-\frac{r_{g}}{2},\frac{r_{g}}{2}\right]$. By first-order Lagrangian approximation, the steering vector of the *k*th signal can be approximated as $\tilde{a}\left(\theta_{k}\right)\approx \tilde{a}\left({\hat{\theta }}_{k}^{\left(1\right)}\right)+\tilde{b}\left({\hat{\theta }}_{k}^{\left(1\right)}\right)\left(\theta_{k}-{\hat{\theta }}_{k}^{\left(1\right)}\right)$. Consequently, the off-grid model can be denoted as

(27) $$\tilde{z}\;=\;\left(\tilde{A}+\tilde{B}\Delta \right)\tilde{p}+\sigma^{2}1_{3MN+M-N},$$

where $\tilde{A}\;=\;\left[\tilde{a}\left({\hat{\theta }}_{1}^{\left(1\right)}\right),\tilde{a}\left({\hat{\theta }}_{2}^{\left(1\right)}\right),\cdots ,\tilde{a}\left({\hat{\theta }}_{K}^{\left(1\right)}\right)\right]\in C^{\left(3MN+M-N\right)\times K}$ is the array manifold matrix for extended virtual array and $\tilde{a}\left({\hat{\theta }}_{k}^{\left(1\right)}\right)$ is the steering vector for the grid point closest to the *k*-th signal with the *i*-th element $e^{j\pi {\bar{d}}_{i}\sin{\hat{\theta }}_{k}^{\left(1\right)}}$, ${\bar{d}}_{i}\in \bar{D}$. $\tilde{B}\;=\;\tilde{B}\left(\theta_{coarse}\right)\;=\;\left[\tilde{b}\left({\hat{\theta }}_{1}^{\left(1\right)}\right),\tilde{b}\left({\hat{\theta }}_{2}^{\left(1\right)}\right),\cdots ,\tilde{b}\left({\hat{\theta }}_{K}^{\left(1\right)}\right)\right]\in C^{\left(3MN+M-N\right)\times K}$ is the derivative of $\tilde{A}$ with respect to $\theta_{coarse}$. $\tilde{p}\in R^{K\times 1}$ is a nonnegative signal power vector.

Based on (27), a two-step iterative method is proposed for solving the grid biases. The first step is to fix the grid bias vector δ and solve the optimization problem below:

(28) $${\tilde{p}}^{\left(j+1\right)}=\arg\min_{\tilde{p}\geq 0}{∥\tilde{p}∥}_{1}\;\;\;\;\;\;\;\;\;\;\;\;\;\;\;s.t.\;\;\;\;\;\;\;\;\;\;\;\;\;\;\;\;\;{∥\tilde{z}\;-\;\left(\tilde{A}+\tilde{B}\Delta^{\left(j\right)}\right)\tilde{p}∥}_{2}\leq \eta_{0}.$$

The unconstrained optimization problem [38] of (28) can be expressed as

(29) $${\tilde{p}}^{\left(j+1\right)}=\arg\min_{\tilde{p}\geq 0}\left[\frac{1}{2}\;{∥\tilde{z}\;-\;\left(\tilde{A}+\tilde{B}\Delta^{\left(j\right)}\right)\tilde{p}∥}_{2}+\eta_{0}{∥\tilde{p}∥}_{1}\right],$$

where $\eta_{0}$ is a regularization parameter that can be adjustable to trade off the sparsity of $\tilde{p}$ and the least-squares error between the observations and estimators. The superscript $\left(j+1\right)$ represents the $\left(j+1\right)$-th iteration.

The optimization problem in (29) can be solved by CVX toolbox [39]. Then, turning to the second step, fix the obtained nonnegative vector ${\tilde{p}}^{\left(j+1\right)}$ and update the grid bias vector δ by

(30) $$\delta^{\left(j+1\right)}=\arg\min_{\delta }{∥\tilde{z}\;-\;\left(\tilde{A}+\tilde{B}\Delta \right){\tilde{p}}^{\left(j+1\right)}∥}_{2}^{2}.$$

To solve the optimization problem about δ, we first expand (30) as

(31) $$\begin{array}{c} {∥\tilde{z}\;-\;\left(\tilde{A}+\tilde{B}\Delta \right){\tilde{p}}^{\left(j+1\right)}∥}_{2}^{2}\\ ={\left(\tilde{z}\;-\;\tilde{A}{\tilde{p}}^{\left(j+1\right)}\right)}^{H}\left(\tilde{z}\;-\;\tilde{A}{\tilde{p}}^{\left(j+1\right)}\right)-{\left(\tilde{z}\;-\;\tilde{A}{\tilde{p}}^{\left(j+1\right)}\right)}^{H}\tilde{B}\Delta {\tilde{p}}^{\left(j+1\right)}\\ \;-{\left(\tilde{B}\Delta {\tilde{p}}^{\left(j+1\right)}\right)}^{H}\left(\tilde{z}\;-\;\tilde{A}{\tilde{p}}^{\left(j+1\right)}\right)+{\left(\tilde{B}\Delta {\tilde{p}}^{\left(j+1\right)}\right)}^{H}\tilde{B}\Delta {\tilde{p}}^{\left(j+1\right)}. \end{array}$$

Removing the terms that are not related to δ in (31) and letting ${\tilde{p}}^{\left(j+1\right)}\;=\;\tilde{p}$, we can obtain

(32) $$\begin{array}{c} -{\left(\tilde{z}\;-\;\tilde{A}\tilde{p}\right)}^{H}\tilde{B}\Delta \tilde{p}-{\left(\tilde{B}\Delta \tilde{p}\right)}^{H}\left(\tilde{z}\;-\;\tilde{A}\tilde{p}\right)+{\left(\tilde{B}\Delta \tilde{p}\right)}^{H}\tilde{B}\Delta \tilde{p}\\ \;=\;-2ℜ\left\{{\left(\tilde{z}\;-\;\tilde{A}\tilde{p}\right)}^{H}\tilde{B}\Delta \tilde{p}\right\}+{\tilde{p}}^{H}\Delta^{H}{\tilde{B}}^{H}\tilde{B}\Delta \tilde{p}\\ =-2ℜ\left\{\mathrm{tr}\left(\tilde{p}{\left(\tilde{z}\;-\;\tilde{A}\tilde{p}\right)}^{H}\tilde{B}\Delta \right)\right\}+\mathrm{tr}\left(\Delta {\tilde{B}}^{H}\tilde{B}\Delta \tilde{p}{\tilde{p}}^{H}\right)\\ =-2ℜ\left\{{\left(\mathrm{diag}\left(\tilde{p}{\left(\tilde{z}\;-\;\tilde{A}\tilde{p}\right)}^{H}\tilde{B}\right)\right)}^{T}\delta \right\}+\delta^{T}\left({\tilde{B}}^{H}\tilde{B}\circ {\left(\tilde{p}{\tilde{p}}^{H}\right)}^{*}\right)\delta . \end{array}$$

For convenience, let $h^{T}={\left(\mathrm{diag}\left(\tilde{p}{\left(\tilde{z}\;-\;\tilde{A}\tilde{p}\right)}^{H}\tilde{B}\right)\right)}^{T}$ and $C={\tilde{B}}^{H}\tilde{B}\circ {\left(\tilde{p}{\tilde{p}}^{H}\right)}^{*}$. Then, Equation (32) can be written as

(33) $$-2ℜ\left(h^{T}\delta \right)+\delta^{T}C\delta =-2ℜ\left(h^{T}\right)\delta +\delta^{T}C\delta .$$

Let the derivative of Equation (33) with respect to δ be equal to zero. Then, we can get the updated grid bias vector $\delta^{\left(j+1\right)}$ as

(34) $$\delta^{\left(j+1\right)}\;=\;ℜ\left(C^{-1}h\right).$$

As described before, the grid bias $\delta_{k}\left(k=1,2,\cdots ,K\right)$ is constrained in $\left[-\frac{r_{g}}{2},\frac{r_{g}}{2}\right]$, so we have

(35) $$\delta_{k}^{\left(j+1\right)}\;=\;\left\{\begin{array}{cc} -\frac{r_{g}}{2} & \delta_{k}^{\left(j+1\right)}<-\frac{r_{g}}{2},\\ \frac{r_{g}}{2} & \delta_{k}^{\left(j+1\right)}>\frac{r_{g}}{2},\\ \delta_{k}^{\left(j+1\right)} & otherwise. \end{array}\right.$$

After the iteration converges, the final estimate of the grid bias vector is obtained and can be denoted as $\delta_{final}$. Consequently, the final DOA estimation results can be expressed as

(36) $$\hat{\theta }\;=\;\theta_{coarse}+\delta_{final}.$$

### 3.3. Summary of the Steps about the Proposed Method

Synthesizing the foregoing analysis, the steps of the proposed method can be summarized as follows:

**Step** **1:** Compute the sample covariance matrix ${\hat{R}}_{xx}$ according to Equation (4);
**Step** **2:** By vectorizing ${\hat{R}}_{xx}$, get $\hat{r}$ according to Equation (8). Then, remove the repeated entries of $\hat{r}$ and sort the remaining entries to get z as Equation (10);
**Step** **3:** Represent z sparsely according to Equation (14) and get the noiseless received signal model according to Equations (15) and (16);
**Step** **4:** Normalize the sample covariance matrix estimation error according to Equations (9), (12), (17) and (18). Then, solve the optimization problem (20) to get $\bar{p}$. The grid points corresponding to *K* maximum values of $\bar{p}$ are the coarse DOAs $\theta_{coarse}$;
**Step** **5:** Get modified sample covariance matrix $\tilde{R}$ according to Equation (26);
**Step** **6:** Vectorize $\tilde{R}$ and construct the off-grid model according to Equation (27);
**Step** **7:** Fix the grid bias vector $\delta^{\left(j\right)}$ and solve the optimization problem (29) to get ${\tilde{p}}^{\left(j+1\right)}$;
**Step** **8:** Fix ${\tilde{p}}^{\left(j+1\right)}$ and update grid bias vector $\delta^{\left(j+1\right)}$ according to Equations (34) and (35);
**Step** **9:** Terminate the iterative process if convergence criteria $∥\delta^{\left(j+1\right)}-\delta^{\left(j\right)}∥/∥\delta^{\left(j\right)}∥\leq \tau$ is satisfied or the number of iterations exceeds the maximum one. Otherwise, return to **Step 7** and continue the iteration process.
**Output:**  The final grid bias vector $\delta_{final}$ and DOA estimation results $\hat{\theta }\;=\;\theta_{coarse}+\delta_{final}$.

## 4. Numerical Simulations

In this section, we perform several simulation experiments to demonstrate the excellent performance of the proposed method for DOA estimation, and also compare it with SS-MUSIC [16], low rank matrix denoising (LRD) [17], LASSO [23] and off-grid sparse Bayesian inference (OGSBI) [28]. In particular, for estimation accuracy comparision, the Cramer–Rao bound (CRB) for coprime arrays [40] is included. The coprime arrays considered here are composed of ten sensors with two coprime integers M=3 and N=5. The positions of sensors in the first subarray are $\left[0,3,6,9,12\right]\frac{\lambda }{2}$ and the others in the second subarray are $\left[0,5,10,15,20,25\right]\frac{\lambda }{2}$. The zeroth sensor is assumed to be the reference element, which is shared by the two subarrays. The scale factor γ in (26) is determined by traversal simulation experiments [41]. The regularization parameter $\eta_{0}$ in (29) is set as 0.3. The iterative convergence criterion is $∥\delta^{\left(j+1\right)}-\delta^{\left(j\right)}∥/∥\delta^{\left(j\right)}∥\leq 10^{-4}$ and the maximum number of iterations is 60. The predefined grid points for LASSO, OGSBI and the coarse estimation process in the proposed method are sampled from $\left[-90^{\circ },90^{\circ }\right]$ with sampling interval being $1^{\circ }$. For fair comparison, the peak search process in SS-MUSIC and LRD is performed with the same step size of $1^{\circ }$ in $\left[-90^{\circ },90^{\circ }\right]$.

### 4.1. Detection Performance

**Figure 2:**
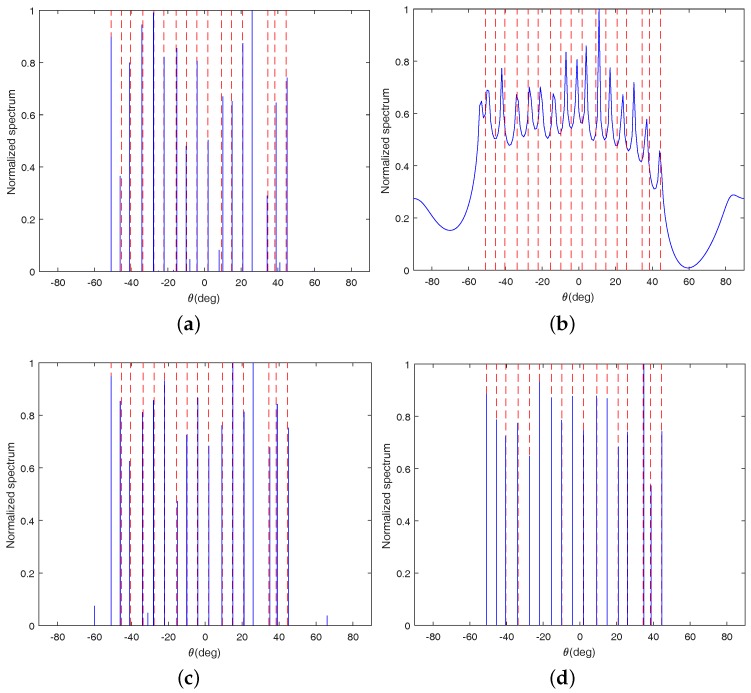
Spatial spectrums of the four algorithms with 17 sources. (**a**) Off-grid sparse Bayesian inference (OGSBI); (**b**) Low rank matrix denoising (LRD); (**c**) Least absolute shrinkage and selection operator (LASSO); (**d**) Proposed method.

In this subsection, the detection performance of the proposed method is compared with that of the methods OGSBI, LASSO and LRD. All of these methods can detect more sources than sensors with using coprime arrays. In theory, LRD can only identify at most 17 sources under the coprime arrays set above. Therefore, for convenience of comparison, we consider 17 randomly distributed narrowband uncorrelated sources here, which are located at $-50.88^{\circ }$, $-45.36^{\circ }$, $-40.35^{\circ }$, $-33.64^{\circ }$, $-27.60^{\circ }$, $-22.12^{\circ }$, $-15.46^{\circ }$, $-9.80^{\circ }$, $-4.18^{\circ }$, $1.80^{\circ }$, $9.23^{\circ }$, $14.74^{\circ }$, $20.80^{\circ }$, $25.96^{\circ }$, $34.46^{\circ }$, $38.39^{\circ }$ and $44.51^{\circ }$. The number of sampled snapshots is 800 and the Signal-to-Noise Ratio (SNR) is set as 0 dB. The scale factor γ is 0.5. The spatial spectra of the four algorithms are shown in Figure 2a–d, respectively. In these figures, the red dashed line indicates the true direction of the incident signal, and the solid blue line indicates the estimated spectral line.

From Figure 2, we can see that OGSBI, LASSO and the proposed method can detect all 17 sources successfully. However, there are still some small spurious peaks in Figure 2a for OGSBI and Figure 2c for LASSO. Furthermore, from Figure 2b, it is easy to find that LRD fail to detect all sources. In its spatial spectrum, several peaks are missed and some of the existing spectral peaks deviate from the true signals a lot. Therefore, it can be concluded that the detection performance of the proposed method is superior to the other three algorithms.

### 4.2. Resolution Ability

In this subsection, the resolution ability of the proposed method is still compared with that of the three algorithms mentioned in Section 4.1. We test the resolution abilities of these algorithms by detecting two closely spaced sources which are located at $-31.25^{\circ }$ and $-29.64^{\circ }$, respectively. Here, the SNR is set as 0 dB and the number of sampled snapshots is set as 800. Additionally, the scale factor γ is identical with that in Section 4.1.

**Figure 3:**
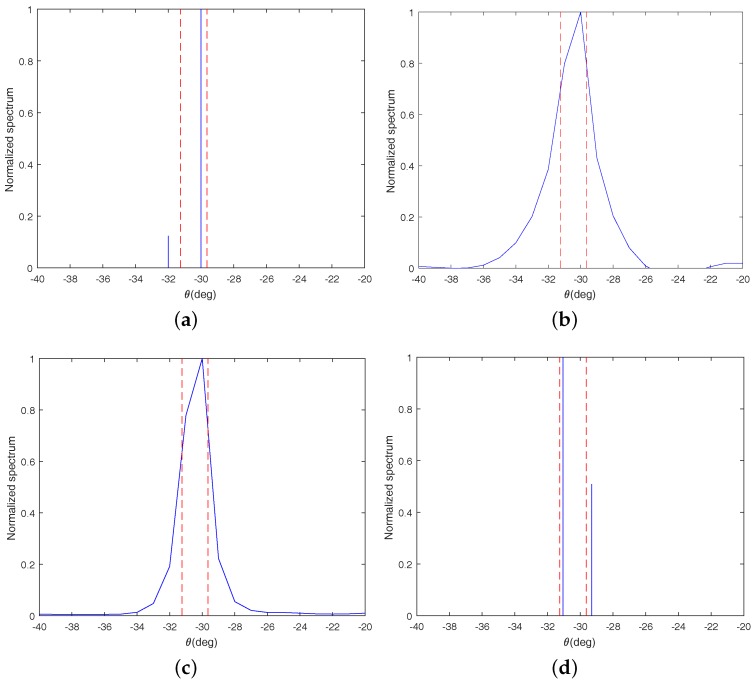
Resolution ability comparision for proposed method and other three algorithms with two closely spaced sources. (**a**) OGSBI; (**b**) LRD; (**c**) LASSO; (**d**) Proposed method.

From Figure 3, it is obvious to find that there is only one peak in the spatial spectrums for LRD and LASSO, which means that both of them can not identify the two signals successfully. In addition, from Figure 3d, we can find that the proposed method identifies the two signals successfully and its estimated DOAs about the two signals are $-31.05^{\circ }$ and $-29.3^{\circ }$, respectively. Though there are errors between the estimated DOAs and the true DOAs, the errors are very small and within an acceptable range. From Figure 3a, it can be seen that OGSBI can also identify the two signals, but the estimation error for OGSBI is larger than the proposed method. Therefore, it can be concluded that the resolution ability of the proposed method is the best among all four of the algorithms.

### 4.3. Estimation Accuracy

In this subsection, the estimation accuracy of each algorithm is reflected by its root-mean-square error (RMSE), which is defined as

(37) $$\mathrm{RMSE}=\sqrt{\frac{1}{QK}\sum_{q=1}^{Q}\sum_{k=1}^{K}{\left({\hat{\theta }}_{k,q}-\theta_{k}\right)}^{2}},$$

where *Q* is the total number of Monte Carlo trials, ${\hat{\theta }}_{k,q}$ represents the estimate of the *k*-th source in the *q*-th trial. For each scenario, we conduct 1000 Monte Carlo trials to get the corresponding RMSE. The estimation accuracy of the proposed method is compared with that of SS-MUSIC, LRD, LASSO, OGSBI and CRB. Although SS-MUSIC and LRD can theoretically identify at most 17 sources, in reality, the peak leakage phenomenon often occurs when applying them to estimate 16 or 17 sources impinging on the coprime arrays. Therefore, we consider 15 narrowband uncorrelated sources that come from the directions of $-55^{\circ }\;+\;\Delta \theta$, $-47^{\circ }\;+\;\Delta \theta$, $-39^{\circ }\;+\;\Delta \theta$, $-31^{\circ }\;+\;\Delta \theta$, $-23^{\circ }\;+\;\Delta \theta$, $-15^{\circ }\;+\;\Delta \theta$, $-7^{\circ }\;+\;\Delta \theta$, $1^{\circ }\;+\;\Delta \theta$, $9^{\circ }\;+\;\Delta \theta$, $17^{\circ }\;+\;\Delta \theta$, $25^{\circ }\;+\;\Delta \theta$, $33^{\circ }\;+\;\Delta \theta$, $41^{\circ }\;+\;\Delta \theta$, $49^{\circ }\;+\;\Delta \theta$ and $57^{\circ }\;+\;\Delta \theta$, where Δθ is chosen randomly from the interval $\left[-0.5^{\circ },0.5^{\circ }\right]$ in each trial to remove the possible prior information contained in the predefined direction set in the coarse estimation process.

**Figure 4:**
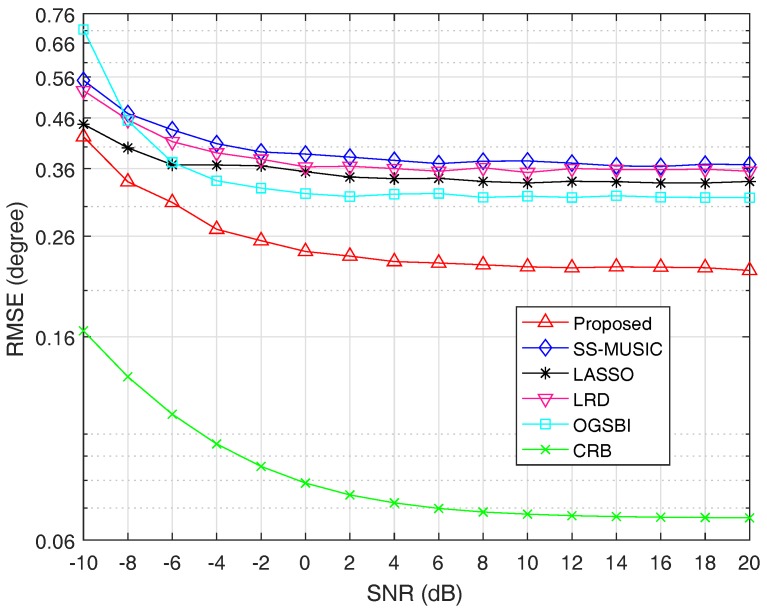
Root-mean-square error (RMSE) as a function of signal-to-noise ratio (SNR) with T=800.

In the first experiment of this subsection, we fix the number of snapshots at 800 and vary SNR from −10dB to 20dB. When SNR is less than or equal to 2dB, γ is set as 0.5. Otherwise, γ is set as 0.7. Figure 4 shows the RMSE curves of the five algorithms and CRB as a function of SNR, respectively.

From Figure 4, we can see that the estimation accuracy is improved with the increase of SNR for all algorithms. It is obvious that the estimation performance of the proposed method outperforms the other four methods. The reason is that spatial smoothing technique causes the extended DOF loss for SS-MUSIC and LRD. Though LASSO can utilize all of the extended DOFs, the assumption that incident signals must fall on the predefined grid points causes its estimation performance degradation. The algorithm OGSBI needs to update the noise variance in each iteration, which makes it sensitive to the noise. In addition, the covariance matrix estimation error and the correlation terms between signal and noise vectors are not considered in OGSBI, which is another reason that the proposed method has better estimation performance than OGSBI.

**Figure 5:**
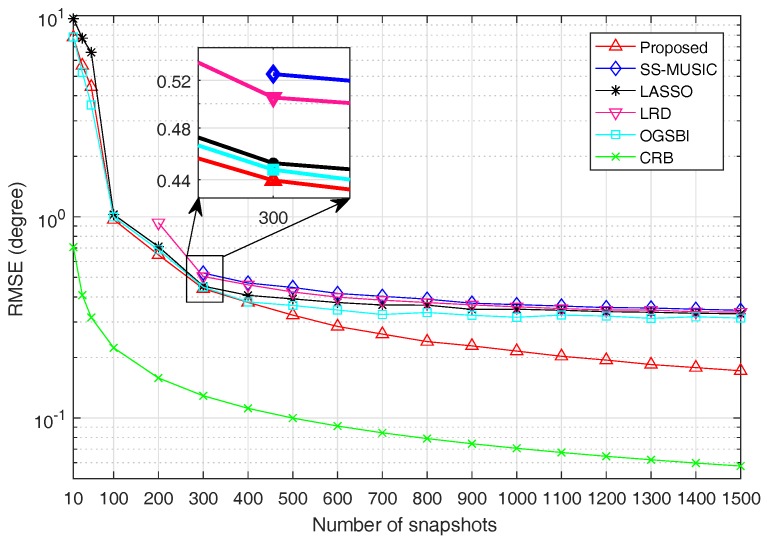
RMSE as a function of snapshots with SNR=0dB.

For the second experiment, the SNR is fixed at 0dB and RMSE is a function of snapshots, the number of which varies from 10 to 1500. When the number of snapshots is less than or equal to 800, γ is set as 0.5. Otherwise, γ is set as 0.8. The RMSE values for all algorithms and CRB versus snapshots are shown in Figure 5.

It is obvious from Figure 5 that the estimation accuracy is improved with the increase of snapshots for all algorithms. For the same reason as described above, the estimation performance of SS-MUSIC, LRD, LASSO and OGSBI is inferior to the proposed method. It can be seen from Figure 5 that when the number of snapshots changes from 10 to 1500, the estimation accuracy of the proposed method is getting better and better than the other four algorithms and finally outperforms the other four algorithms notably by a large margin.

In addition, to have an intuitive understanding on the computation complexity, we compute the average execution time of each algorithm on an Intel Core i7-7700@3.6GHz, 16G RAM PC. Under the same conditions, it takes 0.006 s for SS-MUSIC, 0.670 s for LRD, 0.285 s for LASSO, 1.343 s for OGSBI and 1.599 s for the proposed method. It can be found that SS-MUSIC has the shortest running time. The algorithm OGSBI and the proposed method are a little time-consuming. However, it is worth noting that the simulation results show that the estimation performance of the proposed method is the best among all five of the algorithms. According to the characteristics of each algorithm, they can be applied in different scenarios, respectively. When applied in scenarios where the real-time demand is not very high but the estimation accuracy demand is high, the proposed method is the preferred choice. Conversely, the other several comparison algorithms can be used in the scenarios with high real-time requirements but low estimation accuracy requirements. As we all know, the trade-off between the estimation accuracy and the computation complexity has always been an intractable problem for nearly all algorithms. Therefore, reducing the computation complexity on the basis of the proposed method will be a research direction for us in the future.

## 5. Conclusions

In this paper, we have proposed a sparse-based off-grid DOA estimation method for coprime arrays. By using coprime arrays, we can obtain extended virtual array aperture and increased DOFs. Accordingly, the proposed method can detect more sources than sensors. In practical applications, the existence of the estimation error between the sample and actual covariance matrix is inevitable due to finite snapshots. According to the statistical property of the vectorized covariance matrix estimation error, we construct an optimization problem about the signal power vector in the coarse estimation process so as to obtain the positions of the grid points closest to the true DOAs. In the fine estimation process, we first remove the undesirable correlation terms between the signal and noise vectors to get the modified sample covariance matrix. Then, for solving the grid mismatch problem, an off-grid model with vectorized modified sample covariance matrix is constructed. By applying the two-step iterative method to the off-grid model, the grid biases can be estimated precisely. Combining the coarse estimation results with the estimated grid biases, the final DOA estimation results can be accurately acquired. By considering the covariance matrix estimation error and the correlation terms between signal and noise vectors, the proposed method can achieve better DOA estimation performance than those traditional algorithms that do not consider the finite snapshots effect. In addition, the introduction of an off-grid model solves the grid mismatch problem existing in the traditional on-grid model successfully. Numerical simulation experiments confirmed the superiority and effectiveness of the proposed method.
